# Apolipoprotein E genotype is associated with island sign in lobar intracerebral hemorrhage

**DOI:** 10.3389/fneur.2025.1540307

**Published:** 2025-02-20

**Authors:** Qiong Yang, Haixin Sun, Xinran Ma, Lu Tang, Xiaolu Liu, Xin Huang, Xiao Huang, Yong Chen, Danyang Tian, Xiangzhu Zeng, Nan Li, Wenzhi Wang, Dongsheng Fan

**Affiliations:** ^1^Department of Neurology, Peking University Third Hospital, Beijing, China; ^2^Beijing Neurosurgical Institute, Beijing Tiantan Hospital, Capital Medical University, Beijing, China; ^3^Beijing Municipal Key Laboratory of Clinical Epidemiology, Beijing, China; ^4^Department of Radiology, Peking University Third Hospital, Beijing, China; ^5^School of Public Health Department of Epidemiology and Biostatistics, Peking University, Beijing, China; ^6^Beijing Key Laboratory of Biomarker and Translational Research in Neurodegenerative Diseases, Beijing, China; ^7^Key Laboratory for Neuroscience, National Health Commission/Ministry of Education, Peking University, Beijing, China

**Keywords:** APOE genotype, island sign, intracerebral hemorrhage, genetics, cerebral amyloid angiopathy

## Abstract

**Background:**

The island sign is a predictor of hematoma expansion and worse outcomes in patients of spontaneous primary intracerebral hemorrhage (ICH). The biological mechanism of the island sign remains unclear, but its presence might be influenced by the underlying vasculopathy related to Apolipoprotein E (*APOE*) genotypes. Therefore, we aimed to research the association between *APOE* genotypes and the island sign.

**Methods:**

We enrolled patients with primary supratentorial ICH in a multicenter cohort in northern China with baseline noncontrast CT images performed within 14 days after symptoms onset and *APOE* genotype available. The island sign was rated on the CT images according to validated criteria. Univariable and multivariable analyses were used to identify the association between *APOE* genotypes and the island sign, stratified by the ICH location.

**Results:**

Among 460 patients enrolled, 122 were lobar ICH. In all patients, after adjusting for age, sex, hypertension, and time to CT, the presence of the *APOE* ε4 allele (OR 2.020, 95% CI 1.064–3.834, *p* = 0.032) was associated with the island sign, whereas the presence of the *APOE* ε2 allele (OR 0.734, 95% CI 0.339–1.593, *p* = 0.435) was not. After stratifying by ICH location, multivariable analysis revealed that *APOE* ε4 (OR 3.510, 95% CI 1.393–8.846, *p* = 0.008), rather than ε2 (OR 0.621, 95% CI 0.203–1.901, *p* = 0.404), was associated with the island sign in lobar ICH patients. Neither the ε2 nor the ε4 allele was associated with the island sign among nonlobar ICH patients.

**Conclusion:**

The *APOE* ε4 allele was associated with the island sign in lobar ICH patients. Our findings indicate that the presence of the island sign may be influenced by the underlying vasculopathy related to *APOE* ε4, which increases amyloid deposition in the cerebral vasculature.

## 1 Introduction

Intracerebral hemorrhage (ICH) comprises 10–15% of all strokes worldwide. This severe form of stroke has an early-term mortality of approximately 30–40% (1). A meta-analysis revealed an overall incidence of ICH of 24.6 per 100,000 person-years (2, 3) and that Asian populations are twice as likely to experience ICH as white populations (2, 3). Given the growing aging population and the widespread use of anticoagulants, the ICH incidence is expected to remain substantial, despite ongoing public health efforts to improve hypertension management (1).

Hematoma expansion (HE) prevails in 20% of ICH patients and predicts worse outcomes (4). Preventing HE appears to be an appealing therapeutic strategy, but how to early identify high risk patients when they present with ICH remains challenging. Previous reports proposed imaging predictors for identifying hematomas that have the potential to expand, such as the spot sign observed in CT angiography and several noncontrast CT features including the island sign (5, 6). The island sign, characterized by multifocal small bleeding around the main hematoma, can reflect a hematoma with an extremely irregular shape (7–9). The exact mechanisms underlying the formation of the island sign remain unclear; one explanation is that as the main hematoma, which represents rupture and bleeding of a single blood vessel, expands, active bleeding from adjacent arterioles may cause island-like hematomas, forming the island sign (7). Another explanation is that the island sign may be caused by rupture of several arterioles leading to multifocal active bleeding (7).

The apolipoprotein E (*APOE*) gene is an important genetic risk factor for ICH (10, 11). Previous studies revealed that the presence of *APOE* ε2 and ε4 increases the risk of lobar ICH (12–14). Moreover, *APOE* ε2 is linked with larger ICH volumes (15), hematoma expansion (16) and the presence of CTA spot signs (17) in lobar ICH patients; *APOE* ε4 is associated with functional dependency and poor survival after ICH (10); and both ε2 and ε4 are associated with a greater risk of ICH recurrence (10).

The underlying mechanism by which APOE alleles influence ICH may be related to their effects on cerebral amyloid angiopathy (CAA). CAA, defined by the deposition of beta-amyloid proteins in the walls of small cortical and leptomeningeal vessels in the brain (18), is an important cause of lobar ICH in elders (19). The *APOE* ε4 allele is an established risk factor for CAA (18). The presence of *APOE* ε4 enhances the severity of amyloid deposition in the cerebral vasculature which may accelerate the vascular damage and cause vascular rupture, whereas *APOE* ε2 is predominantly related to the rupture and bleeding of these amyloid-laden vessels (20). Besides, previous studies (21–25) and meta-analyses (26) have shown that irregular borders are among the most common imaging features of CAA related ICH, though the feature has not been clearly defined.

Therefore, we conducted a prospective, multicenter study of ICH cohort to test the hypothesis that the *APOE* genotype is associated with the island sign in lobar ICH patients.

## 2 Methods

### 2.1 Study design and participants

Data from a prospective multicenter cohort of acute primary ICH patients who were recruited from 19 hospitals across Beijing, Hebei, and Inner Mongolia in northern China between 2015 and 2019 were analyzed. The main inclusion criteria were as follows: (1) primary spontaneous supratentorial ICH and (2) available *APOE* genotype data. Patients were excluded if they had any of the following characteristics: (1) secondary ICH due to vascular malformation, tumor, trauma or hemorrhagic cerebral infarction, etc.; (2) no noncontrast CT scan performed within 14 days after the onset of symptoms or low-quality images; (3) head surgery performed before the baseline CT scan; or (4) an unknown exact time of onset (to the minute).

This cohort study was approved by the Ethics Committee of Peking University Third Hospital [(2014)-191-3] (Clinical Trial Registry on clinicaltrials.gov, NCT02361411). Informed consent in writing was obtained from all patients or their representatives.

### 2.2 Data collection

Individual patient data, including age, sex, vascular risk factors, history of previous ICH, and medication history, were systematically and prospectively collected and documented by trained neurologists at the time of the index symptomatic ICH, based on medical records or information provided by patients or their relatives. The National Institutes of Health Stroke Scale (NIHSS) score was assessed upon admission. Medical records were reviewed to obtain the time to initial CT imaging.

### 2.3 Image analysis

CT images were examined by trained study personnel to identify the location and volume of the ICH and to assess for the presence of intraventricular hemorrhage (IVH). The ICH location was defined as supratentorial (lobar or nonlobar) or infratentorial (cerebellum or brainstem) based on the Cerebral Hemorrhage Anatomical RaTing Instrument (CHARTS) (27). The volume of the ICH was calculated from the baseline CT images with the ABC/2 method.

**Figure 1:**
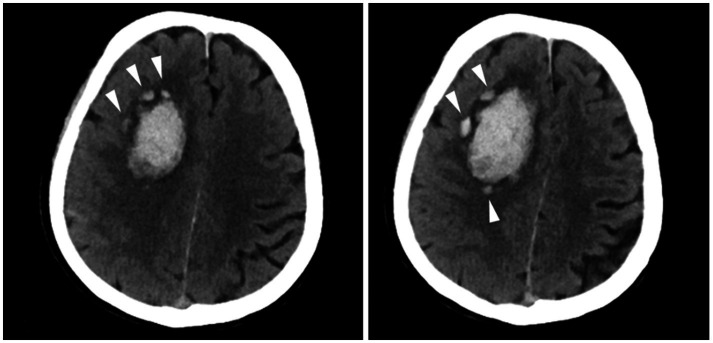
Examples of the island sign. A patient with the island sign (three or more small, scattered hematomas separate from the main hematoma).

The definition of island sign was (1) the presence of three or more small, scattered hematomas separate from the main hematoma or (2) the presence of four or more small hematomas, some or all of which might be connected to the main hematoma (7). The presence of the island sign was assessed according to published criteria (7) by 2 experienced investigators (Figure 1). Discrepancies were resolved through consensus after the investigators reviewed all the scans. All researchers evaluating the imaging were blinded to both the clinical information and the patient’s *APOE* genotype.

### 2.4 Genotyping

With DNA extracted from whole blood samples donated by the patient at enrollment, *APOE* gene loci (rs7412 and rs429358) were tested and then, translated to *APOE* genotypes (ε2/ε2, ε3/ε2, ε2/ε4, ε3/ε3, ε3/ε4, and ε4/ε4). Participants carrying the ɛ2/ɛ2, ε3/ε2, and ɛ2/ɛ4 genotypes were defined as *APOE*-ɛ2 carriers, whereas those carrying the ɛ2/ɛ4, ɛ3/ɛ4, or ɛ4/ɛ4 genotype were defined as *APOE*-ɛ4 carriers. All laboratory staffs performing genotyping were blinded to the clinical data and CT images.

### 2.5 Statistical analysis

Categorical variables were presented as counts with percentages (%), while continuous variables were reported as medians with interquartile ranges (IQRs) due to the nonnormal distribution of the data.

Univariable and multivariable logistic regression analyses were conducted to assess the associations between the presence of *APOE* ε2 and/or ε4 alleles and the island sign. Multivariable model 1 included prespecified predictors, which included age, sex, hypertension, time to CT, and the presence of *APOE* ε2 or ε4 allele. Multivariable model 2 included the aforementioned prespecified predictors along with variables that had a *p* value <0.1 in the univariable analysis. We also performed these analyses after stratifying by location, i.e., lobar versus nonlobar ICH. Finally, we conducted subgroup analyses for patients whose time to CT was within 6 h of ICH onset. Statistical significance was defined for *p* < 0.05. All analyses were performed with SPSS (version 26.0).

## 3 Results

### 3.1 Study population

**Figure 2:**
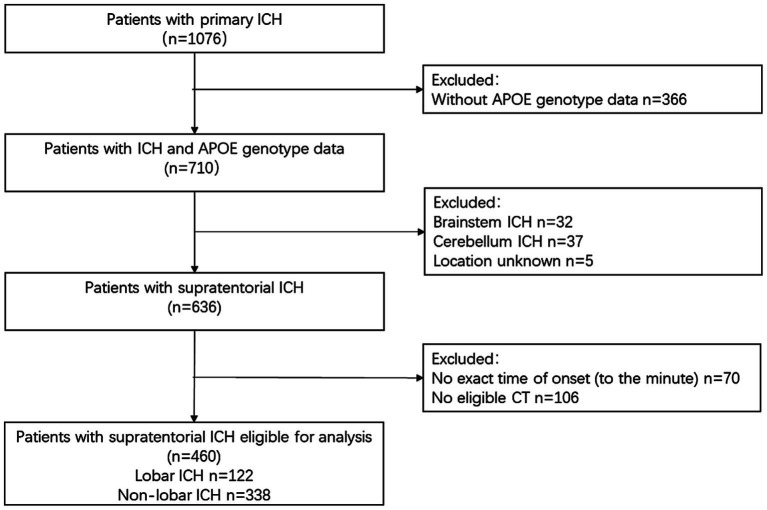
Study flowchart. ICH, intracerebral hemorrhage; *APOE*, apolipoprotein E.

A total of 460 patients were eligible for analysis, with a median age of 60 (51, 73) years, and 297 of them were male (64.6%). Among them, 122 patients had lobar ICH, and 338 patients had nonlobar ICH. Figure 2 shows the flow chart for patient inclusion. The included patients had less hypertension, less diabetes mellitus, lower percentage of moderate to severe alcohol consumption, and shorter time to CT than excluded patients (Supplementary Table S1).

The baseline characteristics of all patients stratified by ICH location were shown in Table 1. Patients in the lobar ICH group were older and had less hypertension, but had a greater ICH volume and a higher percentage of previously ICH, island sign and *APOE* ε4 carriers, compared to those in the nonlobar ICH group (Table 1). Specifically, the island sign was presented in 32 (26.2%) and 31 (9.2%) of patients in the lobar and nonlobar ICH groups, respectively, while 30 patients (24.6%) in the lobar group and 51 patients (15.1%) in the nonlobar group were *APOE* ε4 carriers.

**Table 1:** Baseline characteristics of patients with ICH (all, lobar, and nonlobar).

Variable	All ICH (*n* = 460)	Lobar ICH (*n* = 122)	Nonlobar ICH (*n* = 338)	*p* value
Demographics				
Age, median (IQR)	60 (51,73)	71 (56,79)	58 (50,67)	<0.001
Male, *N* (%)	297/460 (64.6)	75/122 (61.5)	222/338 (65.7)	0.405
Risk factors				
Hypertension, *N* (%)	310/450 (68.9)	65/122 (53.3)	245/328 (74.7)	<0.001
Diabetes mellitus, *N* (%)	61/451 (13.5)	20/121 (16.5)	41/330 (12.4)	0.260
Current smoking, *N* (%)	109/439 (24.8)	29/114 (25.4)	80/325 (24.6)	0.462
Moderate to severe alcohol consumption, *N* (%)	40/451 (8.9)	7/118 (5.9)	33/333 (9.9)	0.196
Previous ICH, *N* (%)	56/450 (12.4)	24/120 (20.0)	32/330 (9.7)	0.004
Previous OAC use, *N* (%)	2/443 (0.5)	1/115 (0.9)	1/328 (0.3)	0.458
Previous antiplatelet use, *N* (%)	45/426 (10.6)	13/110 (11.8)	32/316 (10.1)	0.619
ICH data				
NIHSS score^a^, median (IQR)	7 (2, 13)	2 (1,12)	9 (3,13)	0.117
Time to CT, h, median (IQR)	6.7 (2.7, 25.0)	12.8 (4.1, 47.6)	5.6 (2.5, 21.1)	0.053
ICH volume, median (IQR)	11.7 (4.7, 25.1)	22.7 (11.6, 44.4)	8.4 (4.0, 18.7)	<0.001
Intraventricular hemorrhage, *N* (%)	147/460 (32.0)	35/122 (28.7)	112/338 (33.1)	0.367
Island sign, *N* (%)	63/460 (13.7)	32/122 (26.2)	31/338 (9.2)	<0.001
*APOE* ε2 allele, *N* (%)	85/460 (18.5)	27/122 (22.1)	58/338 (17.2)	0.226
*APOE* ε4 allele, *N* (%)	81/460 (17.6)	30/122 (24.6)	51/338 (15.1)	0.019

^a85 patients with missing data. ICH, intracerebral hemorrhage; IQR, interquartile range; OAC, oral anticoagulant; NIHSS, National Institutes of Health Stroke Scale; APOE, apolipoprotein E.^

### 3.2 Predictors of the island sign on CT scan performed within 14 days of ICH onset

#### 3.2.1 All ICH

In the univariable analysis, greater age (OR 1.026, 95% CI 1.006–1.046, *p* = 0.011), the presence of hypertension (OR 0.477, 95% CI 0.275–0.825, *p* = 0.008), greater ICH volume (OR 1.040, 95% CI 1.029–1.051, *p* < 0.001), the presence of IVH (OR 1.731, 95% CI 1.006–2.980, *p* = 0.048) and the presence of the *APOE* ε4 allele (OR 1.923, 95% CI 1.037–3.565, *p* = 0.038) were associated with the presence of the island sign (Table 2).

**Table 2:** Univariable analysis for the presence of the island sign.

Variable	All ICH (*n* = 460)		Lobar ICH (*n* = 122)		Nonlobar ICH (*n* = 338)	
	OR (95% CI)	*P* value	OR (95% CI)	*P* value	OR (95% CI)	*P* value
Age	1.026 (1.006–1.046)	0.011	1.006 (0.977–1.035)	0.703	1.022 (0.994–1.050)	0.121
Sex (male versus female)	1.026 (0.588–1.792)	0.927	1.273 (0.548–2.956)	0.575	0.945 (0.436–2.046)	0.886
Hypertension^a^	0.477 (0.275–0.825)	0.008	0.595 (0.264–1.342)	0.211	0.614 (0.273–1.379)	0.237
Diabetes mellitus^b^	0.651 (0.267–1.583)	0.343	0.263 (0.057–1.205)	0.085	1.094 (0.361–3.310)	0.874
Current smoking^c^	0.945 (0.678–1.316)	0.737	1.079 (0.658–1.769)	0.762	0.797 (0.489–1.297)	0.361
Moderate to severe alcohol consumption^d^	1.765 (0.771–4.041)	0.179	2.452 (0.515–11.669)	0.260	1.964 (0.697–5.535)	0.201
Previous ICH^e^	0.724 (0.297–1.769)	0.479	0.330 (0.091–1.194)	0.091	1.038 (0.297–3.634)	0.953
Previous OAC use^f^	7.167 (0.442–116.260)	0.166	-^h^	-^h^	-^h^	-^h^
Previous antiplatelet use^g^	0.452 (0.135–1.511)	0.197	0.497 (0.103–2.392)	0.383	0.320 (0.042–2.442)	0.272
Time to CT, h, median	0.997 (0.991–1.003)	0.338	0.996 (0.988–1.004)	0.352	0.994 (0.984–1.005)	0.301
ICH volume, median	1.040 (1.029–1.051)	<0.001	1.042 (1.022–1.061)	<0.001	1.034 (1.020–1.048)	<0.001
Presence of intraventricular hemorrhage	1.731 (1.006–2.980)	0.048	1.440 (0.606–3.425)	0.409	2.344 (1.113–4.936)	0.025
*APOE* ε2	0.810 (0.394–1.666)	0.567	0.758 (0.275–2.089)	0.592	0.694 (0.233–2.065)	0.512
*APOE* ε4	1.923 (1.037–3.565)	0.038	3.597 (1.487–8.699)	0.004	0.578 (0.169–1.978)	0.383

^a10 patients with missing data. b9 patients with missing data. c21 patients with missing data. d9 patients with missing data. e10 patients with missing data. f17 patients with missing data. g34 patients with missing data. hUnivariate logistic regression failed to converge. ICH, intracerebral hemorrhage; IQR, interquartile range; OAC, oral anticoagulant; NIHSS, National Institutes of Health Stroke Scale; APOE, apolipoprotein E.^

In the multivariable analysis, after adjusting for prespecified predictors such as age, sex, hypertension and time to CT, the presence of the *APOE* ε4 allele (OR 2.020, 95% CI 1.064–3.834, *p* = 0.032), but not the presence of the *APOE* ε2 allele (OR 0.734, 95% CI 0.339–1.593, *p* = 0.435), was associated with the island sign (Table 3). Moreover, after adjusting both for the prespecified predictors and variables with *p* < 0.1 in the univariable analysis, including age, sex, hypertension, ICH volume, IVH, and time to CT, the presence of *APOE* ε4 (OR 2.114, 95% CI 1.038–4.307, *p* = 0.039), but not the presence of *APOE* ε2 (OR 0.608, 95% CI 0.252–1.467, *p* = 0.268), was associated with the island sign (Table 3).

**Table 3:** Multivariable analysis for the presence of the island sign.

Variable	All ICH (*n* = 460)		Variable	Lobar ICH (*n* = 122)		Variable	Nonlobar ICH (*n* = 338)	
	OR (95% CI)	*P* value		OR (95% CI)	*P* value		OR (95% CI)	*P* value
Model 1^a,b^			Model 1^a^			Model 1^a,b^		
Age	1.026 (1.005–1.047)	0.013	Age	1.009 (0.977–1.042)	0.580	Age	1.021 (0.992–1.051)	0.155
Sex (male versus female)	1.237 (0.681–2.246)	0.485	Sex (male versus female)	1.621 (0.640–4.105)	0.308	Sex (male versus female)	1.057 (0.461–2.422)	0.896
Hypertension	0.547 (0.312–0.962)	0.036	Hypertension	0.723 (0.298–1.755)	0.474	Hypertension	0.629 (0.276–1.432)	0.269
APOE ε2	0.734 (0.339–1.593)	0.435	APOE ε2	0.621 (0.203–1.901)	0.404	APOE ε2	0.577 (0.166–2.002)	0.386
APOE ε4	2.020 (1.064–3.834)	0.032	APOE ε4	3.510 (1.393–8.846)	0.008	APOE ε4	0.673 (0.193–2.351)	0.535
Time to CT	0.998 (0.992–1.003)	0.420	Time to CT	0.998 (0.989–1.006)	0.617	Time to CT	0.995 (0.985–1.006)	0.414
Model 2^b,c^			Model 2^c,d^			Model 2^b,c^		
Age	1.020 (0.998–1.043)	0.075	Age	1.014 (0.976–1.054)	0.472	Age	1.021 (0.991–1.053)	0.167
Sex (male versus female)	1.115 (0.571–2.174)	0.750	Sex (male versus female)	1.476 (0.463–4.705)	0.510	Sex (male versus female)	0.981 (0.400–2.406)	0.966
Hypertension	0.920 (0.476–1.778)	0.803	Hypertension	1.592 (0.540–4.696)	0.399	Hypertension	0.825 (0.332–2.048)	0.678
APOE ε2	0.608 (0.252–1.467)	0.268	APOE ε2	0.320 (0.070–1.463)	0.142	APOE ε2	0.638 (0.174–2.338)	0.498
APOE ε4	2.114 (1.038–4.307)	0.039	APOE ε4	3.605 (1.152–11.279)	0.028	APOE ε4	1.003 (0.275–3.655)	0.996
Time to CT	1.001 (0.995–1.007)	0.748	Time to CT	1.001 (0.993–1.011)	0.746	Time to CT	0.999 (0.989–1.009)	0.868
ICH volume	1.040 (1.028–1.053)	<0.001	Diabetes mellitus	0.092 (0.011–0.794)	0.030	ICH volume	1.033 (1.017–1.050)	<0.001
IVH	0.982 (0.504–1.913)	0.957	Previous ICH	0.107 (0.019–0.610)	0.012	IVH	1.273 (0.523–3.097)	0.595
			ICH volume	1.049 (1.024–1.073)	<0.001			

^aPrespecified plausible predictors were included. b10 patients were excluded because of missing data. cPrespecified plausible predictors as well as variables with a P value < 0.1 in univariable regression were included. d3 patients were excluded because of missing data. ICH, intracerebral hemorrhage; APOE, apolipoprotein E; IVH, Intraventricular hemorrhage.^

#### 3.2.2 Lobar ICH

In the univariable analysis, a larger ICH volume (OR 1.042, 95% CI 1.022–1.061, *p* < 0.001) and the presence of the *APOE* ε4 allele (OR 3.597, 95% CI 1.487–8.699, *p* = 0.004) were associated with the presence of the island sign (Table 2).

In the multivariable analysis, after adjusting for the prespecified predictors such as age, sex, hypertension and time to CT, the presence of the *APOE* ε4 allele (OR 3.510, 95% CI 1.393–8.846, *p* = 0.008), but not the presence of the *APOE* ε2 allele (OR 0.621, 95% CI 0.203–1.901, *p* = 0.404), was associated with the island sign (Table 3). Moreover, after adjusting for the prespecified predictors and variables with *p* < 0.1 in the univariable analysis, including age, sex, hypertension, diabetes mellitus, previous ICH, ICH volume and time to CT, the presence of the *APOE* ε4 allele (OR 3.605, 95% CI 1.152–11.279, *p* = 0.028), but not that of the *APOE* ε2 allele (OR 0.320, 95% CI 0.070–1.463, *p* = 0.142), was associated with the island sign (Table 3).

#### 3.2.3 Nonlobar ICH

According to the univariable analysis, greater ICH volume (OR 1.034, 95% CI 1.020–1.048, *p* < 0.001) and the presence of IVH (OR 2.344, 95% CI 1.113–4.936, *p* = 0.025) were associated with the island sign (Table 2).

In the multivariable analysis, after adjusting for prespecified predictors, including age, sex, hypertension and time to CT, the presence of neither *APOE* ε4 (OR 0.673, 95% CI 0.193–2.351, *p* = 0.535) nor *APOE* ε2 (OR 0.577, 95% CI 0.166–2.002, *p* = 0.386) was associated with the island sign (Table 3). Moreover, the presence of neither *APOE* ε4 (OR 1.003, 95% CI 0.275–3.665, *p* = 0.996) nor *APOE* ε2 (OR 0.638, 95% CI 0.174–2.338, *p* = 0.498) was associated with the island sign after adjusting for the prespecified predictors and variables with *p* < 0.1 in the univariable analysis, including age, sex, hypertension, ICH volume, IVH and time to CT (Table 3).

### 3.3 Predictors of the island sign on CT scan performed within 6 h of ICH onset

In the univariable analysis, the presence of neither *APOE* ε4 (OR 1.648, 95% CI 0.611–4.447, *p* = 0.324) nor *APOE* ε2 (OR 1.703, 95% CI 0.663–4.373, *p* = 0.269) was associated with the island sign in all ICH patients (Supplementary Table S2); similar results were obtained in the multivariable analysis (Supplementary Table S3). Subgroup analyses for lobar and nonlobar ICH patients also failed to uncover an association between the presence of either *APOE* ε4 or ε2 allele and the presence of the island sign (Supplementary Tables S2, S3).

## 4 Discussion

Our findings demonstrated the association between *APOE* ε4 and the island sign on CT imaging in patients with lobar ICH rather than nonlobar ICH. However, no association between *APOE* ε2 and the island sign was observed in either lobar or nonlobar ICH patients.

Our study revealed for the first time that the *APOE* ε4 allele was associated with the island sign in lobar ICH patients. Though the pathophysiological mechanism remains unclear, the role of *APOE* in the island sign might be consistent with *APOE* in CAA and ICH. The histopathologic mechanisms of *APOE* ε4 and ε2 appear different in CAA. *APOE* ε4 allele increases the deposition of amyloid protein in the wall of small cortical and meningeal vessels, making it vulnerable to rupture, whereas *APOE* ε2 mainly causes blood vessels with amyloid deposition to rupture and bleed (20). Vascular damage caused by amyloid deposition, which is accelerated by *APOE* ε4, impacts a substantial portion of the leptomeningeal and cortical arterioles in patients with CAA (28). We hypothesized this damage makes them more susceptible to multifocal bleeding, leading to the formation of the island sign. In addition, the island sign may contribute to some of the ambiguous imaging features of irregular borders in CAA related ICH observed in previous studies (21–26).

Furthermore, we explored the associations between the island sign and *APOE* across different time windows including 14 days and 6 h. Previously, Li et al. proposed that the island sign mainly appears on images taken within 6 h, reflecting early (within 24 h) hematoma expansion (7). Our study showed that the association between ε4 and the island sign was significant within 14 days but not within 6 h of ICH onset, although the OR values were similar. On one hand, the avalanche effect caused by acute cerebral hemorrhage is an important prerequisite for the formation of the island sign, for which the first 6 h constitutes the peak window (7, 29). On the other hand, the *post hoc* analysis of the TICH-2 trial found that in patients with lobar CAA related ICH, the risk of hematoma expansion increased with time from symptom onset, indicating a longer time window of hematoma expansion, which was different from nonlobar ICH and lobar non-CAA related ICH (28). The mechanisms leading to prolonged hematoma expansion in CAA related ICH may be that the bleeding related to CAA originates from leptomeningeal vessels which form an effective collateral network. As a result, the vasoconstrictive response involved in hemostasis may be less effective (28). In addition, the lobar location of the hemorrhage provides more space and reduces the likelihood of tamponade, which can help stop the bleeding, compared to non-lobar locations (28). This also suggest that the time window for the presence of the island sign may exceed 6 h.

Our study did not find a link between *APOE* ε2 and the island sign, though previous reports have indicated that *APOE* ε2 was related to spot sign, as well as greater hematoma volume and hematoma expansion (15–17). Theoretically, APOE ε2 might be expected to be associated with the island sign within the first 6 h in lobar ICH, similar to its association with the spot sign (17). The proposed mechanism may involve APOE ε2 predisposing to additional vessel rupture, leading to hematoma expansion and the formation of the island sign. In the subgroup analysis of our study, we did observe an increased odds ratio of the association between *APOE* ε2 and the island sign in 6 h, but it was statistically insignificant which may be attributed to the limited number of patients imaged during that time frame. Further research is needed to clarify this potential relationship. In contrast, over the longer 14-day time window, we observed a significant association between the island sign and APOE ε4, rather than APOE ε2. This could be due to vascular amyloid changes induced by APOE ε4, which contribute to the formation of multiple small bleedings surrounding the main hematoma over a prolonged period (beyond 6 h), resulting in the island sign.

The strengths of our study included that the use of data from a multicenter prospective cohort and a thorough evaluation of the neuroimaging. This study added the knowledge of effect of *APOE* ε4 genotype on the island sign over a 14-day period in lobar ICH patients, providing insights into the biological mechanisms underlying the island sign. Given the role of APOE ε4 in CAA and its link to increased recurrence risk in ICH patients, the presence of the island sign may also predict a higher likelihood of ICH recurrence which needs to be clarified in future research. Furthermore, with bedside genotyping available, there would be room of optimization of acute management of ICH by the combination use of island sign and the APOE gene, in terms of risk stratification and an early bundle of care focused on blood pressure control (30), individualized anti-coagulation strategy for patients at high thromboembolic risk including atrial fibrillation, venous thromboembolism, etc. (1). Additionally, the island sign can be easily determined on CT scans, which could potentially improve the management strategies and prognostic prediction in the acute setting when APOE genotype test is not available.

### 4.1 Limitations

(1) Our cohort did not have complete MRI data, preventing us from identifying a subgroup of patients with CAA based on the Boston criteria. (2) The limited number of patients with available baseline CT images within 6 h hindered a thorough investigation of the relationship between the *APOE* allele and the island sign. This was partly due to late arrivals at the hospital, referrals from other facilities, and the inability to secure initial CT images from those hospitals. Further research is necessary to more concretely establish the role of *APOE* to validate our findings.

In conclusion, the *APOE* ε4 allele is associated with the presence of the island sign in lobar ICH patients. Given the known effect of *APOE* ε4 on amyloid deposition in the cerebral vasculature, our findings indicate that *APOE* genotype-related vasculopathies may influence the presence of the island sign.

## Data Availability

The datasets presented in this study can be found in online repositories. The names of the repository/repositories and accession number(s) can be found below: https://ngdc.cncb.ac.cn/omix/, OMIX008306.
