# Supplementary material for: Apolipoprotein E genotype is associated with island sign in lobar intracerebral hemorrhage
Source: Front Neurol. 2025 Feb 20;16:1540307. doi: 10.3389/fneur.2025.1540307 (PMC11882416; doi:10.3389/fneur.2025.1540307)
Supplement: Supplementary file 1 [file Table_1.docx]

**Apolipoprotein E Genotype Is Associated with Island Sign in Lobar Intracerebral Hemorrhage**

**Table S1. Characteristics of ICH patients included and excluded.**

| **Variable** | **Excluded (n=616)** | **Included (n=460)** | **P value** |
| --- | --- | --- | --- |
| **Demographics** |  |  |  |
| **Age, median (IQR)** | 61(52,71) | 60(51,73) | 0.674 |
| **Male, N (%)** | 404/606(66.7) | 297/460(64.6) | 0.474 |
| **Risk factors** |  |  |  |
| **Hypertension, N（%）** | 448/598(74.9) | 310/450(68.9) | 0.031 |
| **Diabetes mellitus, N（%）** | 115/593(19.4) | 61/451(13.5) | 0.013 |
| **Smoker, N（%）** | 160/586(27.3) | 109/439(24.8) | 0.388 |
| **Moderate to severe alcohol consumption, N（%）** | 82/592(13.9) | 40/451(8.9) | 0.014 |
| **Previous ICH, N（%）** | 79/595(13.3) | 56/450(12.4) | 0.691 |
| **Previous OAC use, N（%）** | 6/585(1.0) | 2/443(0.5) | 0.313 |
| **Previous antiplatelet use, N（%）** | 67/561(11.9) | 45/426(10.6) | 0.489 |
| **ICH data** |  |  |  |
| **NIHSS score, median (IQR)** | 6(2, 13) | 7(2, 13) | 0.683 |
| **Time to CT, h, median (IQR)** | 6.7(2.2, 36.3) | 6.7(2.7, 25.0) | 0.007 |
| **ICH volume, median (IQR)** | 6.8(2.4, 20.0) | 11.7(4.7, 25.1) | 0.186 |
| **IVH, N（%）** | 112/398(28.1) | 147/460(32.0) | 0.225 |

Abbreviations: ICH = intracerebral hemorrhage; IQR = interquartile range; OAC = oral anticoagulation; NIHSS = National Institutes of Health Stroke Scale; IVH = Intraventricular hemorrhage

**Table S2. Univariable analysis of the presence of the island signs on CT scan performed within 6 hours of ICH onset**.

|  | **All ICH (n=224)** | | **Lobar ICH (n=45)** | | **Nonlobar ICH (n=179)** | |
| --- | --- | --- | --- | --- | --- | --- |
| **Variable** | **OR (95% CI)** | **P value** | **OR (95% CI)** | **P value** | **OR (95% CI)** | **P value** |
| **Age** | 1.026(0.996-1.057) | 0.090 | 0.991(0.942-1.041) | 0.710 | 1.030 (0.991-1.071) | 0.137 |
| **Male sex** | 0.988(0.422-2.308) | 0.977 | 1.263(0.302-5.275) | 0.749 | 0.936(0.318-2.755) | 0.904 |
| **Hypertension^a^** | 0.607(0.255-1.446) | 0.260 | 0.842(0.206-3.438) | 0.811 | 0.662(0.210-2.085) | 0.480 |
| **Diabetes mellitus^b^** | 0.785(0.221-2.794) | 0.709 | 0.375(0.041-3.424) | 0.385 | 1.026(0.216-4.870) | 0.975 |
| **Current smoking^c^** | 1.069(0.639-1.789) | 0.799 | 1.899(0.764-4.720) | 0.167 | 0.831(0.421-1.641) | 0.594 |
| **Moderate to severe alcohol consumption^d^** | 1.950 (0.516-7.371) | 0.325 | 2.357(0.187-29.745) | 0.507 | 1.910(0.385-9.468) | 0.428 |
| **Previous ICH^e^** | 0.504(0.113-2.252) | 0.370 | 0(0-.) | 0.999 | 1.231(0.257-5.900) | 0.795 |
| **Previous OAC use^f^** | -^h^ | -^h^ | -^h^ | -^h^ | -^h^ | -^h^ |
| **Previous antiplatelet use^g^** | 0.361(0.046-2.821) | 0.332 | 0.542(0.056-5.193) | 0.595 | -^h^ | -^h^ |
| **Time to CT** | 0.989(0.752-1.300) | 0.935 | 0.773(0.478-1.250) | 0.294 | 1.080(0.765-1.525) | 0.661 |
| **ICH volume** | 1.036(1.022-1.050) | <0.001 | 1.031(1.009-1.054) | 0.006 | 1.037(1.018-1.056) | <0.001 |
| **Presence of intraventricular hemorrhage** | 1.911(0.828-4.409) | 0.129 | 2.500(0.592-10.555) | 0.212 | 1.880(0.650-5.440) | 0.244 |
| **APOE ε2 genotype** | 1.703(0.663-4.373) | 0.269 | 3.222(0.690-15.039) | 0.137 | 1.073(0.285-4.032) | 0.917 |
| **APOE ε4 genotype** | 1.648(0.611-4.447) | 0.324 | 1.926(0.441-8.417) | 0.384 | 0.943(0.200-4.455) | 0.941 |

**^a^** 5 patients with missing data.

**^b^** 3 patients with missing data.

**^c^** 11 patients with missing data.

**^d^** 5 patients with missing data.

**^e^** 2 patients with missing data.

**^f^** 8 patients with missing data.

**^g^** 13 patients with missing data.

^h^ Univariate logistic regression failed to converge.

Abbreviations: ICH = intracerebral hemorrhage; IQR = interquartile range; OAC = oral anticoagulant; NIHSS = National Institutes of Health Stroke Scale; *APOE* = apolipoprotein E

**Table S3. Multivariable analysis for the presence of the island sign on CT scan performed within 6 hours of ICH onset**.

|  | **All ICH(n=224)** | |  | **Lobar ICH (n=45)** | |  | | **Nonlobar ICH (n=179)** | |  |
| --- | --- | --- | --- | --- | --- | --- | --- | --- | --- | --- |
| **Variable** | OR (95% CI) | P value | **Variable** | OR (95% CI) | P value | **Variable** | | OR (95% CI) | P value |  |
| **Model 1**^a^**^,b^** | | | **Model 1** ^a^ | | | | **Model 1**^a^**^,b^** | | | |
| **Age** | 1.028(0.997-1.061) | 0.081 | **Age** | 0.989(0.934-1.046) | 0.689 | **Age** | | 1.035(0.993-1.078) | 0.104 |  |
| **Sex (male versus female)** | 1.019(0.414-2.506) | 0.967 | **Sex (male versus female)** | 0.830 (0.149-4.631) | 0.832 | **Sex (male versus female)** | | 1.009(0.314-3.238) | 0.988 |  |
| **Hypertension** | 0.691(0.281-1.697) | 0.420 | **Hypertension** | 1.174(0.232-5.941) | 0.846 | **Hypertension** | | 0.638(0.198-2.058) | 0.452 |  |
| **APOE ε2 allele** | 1.519(0.546-4.220) | 0.423 | **APOE ε2 allele** | 3.77(0.630-22.571) | 0.146 | **APOE ε2 allele** | | 0.785(0.161-3.830) | 0.765 |  |
| **APOE ε4 allele** | 1.778(0.630-5.016) | 0.277 | **APOE ε4 allele** | 1.840 (0.346-9.792) | **0.475** | **APOE ε4 allele** | | 1.094(0.219-5.474) | 0.913 |  |
| **Time to CT, h** | 1.001(0.751-1.334) | 0.996 | **Time to CT, h** | 0.787(0.468-1.324) | 0.366 | **Time to CT, h** | | 1.106(0.766-1.597) | 0.592 |  |
| **Model 2^b,c^** | | | **Model 2^c^** | | | | **Model 2^b,c^** | | | |
| **Age** | 1.017(0.982-1.054) | 0.334 | **Age** | 0.982(0.920-1.048) | 0.578 | **Age** | | 1.037(0.990-1.086) | 0.126 |  |
| **Sex (male versus female)** | 0.777(0.271-2.225) | 0.638 | **Sex (male versus female)** | 0.530 (0.066-4.269) | 0.551 | **Sex (male versus female)** | | 0.876(0.227-3.382) | 0.847 |  |
| **Hypertension** | 1.737(0.534-5.654) | 0.359 | **Hypertension** | 3.586(0.409-31.435) | 0.249 | **Hypertension** | | 1.253(0.295-5.318) | 0.760 |  |
| **APOE ε2 allele** | 1.570(0.489-5.044) | 0.448 | **APOE ε2 allele** | 3.741(0.429-32.637) | 0.232 | **APOE ε2 allele** | | 1.419(0.263-7.666) | 0.684 |  |
| **APOE ε4 allele** | 1.850(0.561-6.097) | 0.312 | **APOE ε4 allele** | 1.727(0.244-12.223) | 0.584 | **APOE ε4 allele** | | 2.052(0.352-11.947) | 0.424 |  |
| **ICH volume** | 1.039(1.023-1.055) | <0.001 | **ICH volume** | 1.037(1.009-1.065) | 0.008 | **ICH volume** | | 1.045(1.020-1.070) | <0.001 |  |
| **Time to CT** | 1.036(0.749-1.434) | 0.830 | **Time to CT** | 0.774(0.438-1.367) | 0.377 | **Time to CT** | | 1.228(0.812-1.858) | 0.331 |  |

^a^ Prespecified plausible predictors were included.

^b^ 5 patients were excluded because of missing data.

^c^ Prespecified plausible predictors as well as variables with a P value < 0.1 in univariable regression were included.

Abbreviations: ICH = intracerebral hemorrhage; *APOE* = apolipoprotein E
